# Applications of the Cambridge Structural Database in chemical education^1^

**DOI:** 10.1107/S0021889810024155

**Published:** 2010-08-03

**Authors:** Gary M. Battle, Gregory M. Ferrence, Frank H. Allen

**Affiliations:** a Cambridge Crystallographic Data Centre (CCDC), 12 Union Road, Cambridge CB2 1EZ, UK; b Department of Chemistry, Illinois State University, Normal, IL 61790-4160, USA

**Keywords:** Cambridge Structural Database, crystallographic education, *WebCSD*

## Abstract

The Cambridge Structural Database (CSD) is a vast and ever growing compendium of accurate three-dimensional structures that has massive chemical diversity across organic and metal–organic compounds. For these reasons, the CSD is finding significant uses in chemical education, and these applications are reviewed. As part of the teaching initiative of the Cambridge Crystallographic Data Centre (CCDC), a teaching subset of more than 500 CSD structures has been created that illustrate key chemical concepts, and a number of teaching modules have been devised that make use of this subset in a teaching environment. All of this material is freely available from the CCDC website, and the subset can be freely viewed and interrogated using *WebCSD*, an internet application for searching and displaying CSD information content. In some cases, however, the complete CSD System is required for specific educational applications, and some examples of these more extensive teaching modules are also discussed. The educational value of visualizing real three-dimensional structures, and of handling real experimental results, is stressed throughout.

## Abstract 2

The educational value of three-dimensional crystal structures in the Cambridge Structural Database (CSD) is discussed in the context of practical use cases and the availability of a free teaching subset of the CSD that can be used in conjunction with *WebCSD*, an application that provides internet access to CSD information content.

## 1. Introduction

In June 1988, the *Journal of Chemical Education* (JCE) published a series of eight papers arising from a symposium on teaching crystallography that was held at the 1987 Meeting of the American Crystallographic Association in Austin, Texas. In his contribution, Duax (1988 ▶) described a course for first-year graduate students of pharmacology, medicinal chemistry and biochemistry on how to interpret, evaluate and use information provided by crystal structure determinations. Central to that course were modules on basic crystallographic concepts – lattices, crystal systems, space groups, symmetry (crystallographic and molecular) and crystal packing – and on molecular geometry – molecular dimensions, stereochemistry, conformational analysis, structure representation and visualization, and the relationship between structure and properties. This latter section made significant use of the Cambridge Structural Database (CSD; Allen, 2002 ▶) which, in 1988, recorded some 55 000 small-molecule crystal structures.

In a purely crystallographic teaching context, the CSD and other structural database resources, particularly the Protein Data Bank (PDB; Berman *et al.*, 2000 ▶), have been an integral part of a variety of schools and courses, such as the Intensive Courses in X-ray Crystallography given by the British and American Crystallographic Associations (BCA, ACA) and courses offered by other national groups. Additionally, anecdotal and published evidence indicates that further significant teaching applications of the developing CSD have been made in many universities at both undergraduate and graduate levels, and in some high schools. Much of the impetus for these broader developments comes from practising crystallographers who thereby provide expert introduction to the value of the crystallographic method to undergraduate chemistry and biology students. These students, in their turn, are then aware of the power of crystallography in their own careers, and some of them may be attracted to make the subject their speciality. This route into the subject is commonly followed by many professional crystallographers whose undergraduate roots are, *inter alia*, in chemistry, biology and materials science, and is entirely in line with the white paper *Crystallography Education Policies for the Physical and Life Sciences: Sustaining the Science of Molecular Structure in the 21st Century*, published by the US National Committee on Crystallography in collaboration with the ACA and the International Union of Crystallography (USNC/IUCr/ACA, 2006 ▶). Here, the importance of educating not only professional crystallographers but also the consumers of their results is clearly stressed, and the role of crystallographers in promoting their subject is an obvious part of this consumer education.

Twenty years on from the JCE papers, and with a database that is an order of magnitude larger (>500 000 structures), the CSD has much to offer in a teaching environment and, if we now include general organics, organometallics and metal complexes, the Duax (1988 ▶) course resumé still summarizes the CSD’s areas of educational value rather well. A knowledge of the three-dimensional nature of chemical compounds is absolutely fundamental to every chemist and to many in related disciplines. Without this understanding, concepts such as conformation, stereochemistry, chirality, metal coordination and molecular symmetry cannot be properly assimilated and understood. It is well known that three-dimensional visualizations enhance students’ learning experience, spatial abilities and conceptual understanding (Bodner & Guay, 1997 ▶; Wu & Shah, 2004 ▶; Williamson & Jose, 2008 ▶), yet even today, when high-resolution three-dimensional graphics capabilities are available on every home computer, chemical structures are still taught using quasi-two-dimensional representations. These representations do not convey the levels of comprehension that are opened up to students by the visualization and interactive manipulation of real three-dimensional mol­ecular images in their own personal computing environment.

In addition to the structural knowledge obtained through crystallography, an exposure to real experimental data further enhances the learning experience for students (Prince, 2004 ▶; Handelsman *et al.*, 2004 ▶; DeHaan, 2005 ▶). The experimental errors and statistical variation inherent in directly measured data provide insights into the outputs of experimental science. Because traditional classroom teaching examples instil a bias that chemical structure should be ‘ideal and perfect’, the use of real experimental results prepares students for the realities of research. Finally, it is good for all students of chemistry and associated disciplines to develop a familiarity with the crystallographic method and its results, since crystallography is rarely taught as a sub-discipline at undergraduate or graduate level, but plays a crucial role in all branches of modern chemistry, as well as in structural biology, the pharmaceutical sciences and materials science.

To address this educational potential, the CCDC has in recent years begun an outreach initiative, principally in structural chemistry but also involving the symmetry aspects of the subject. Here, we summarize that initiative, which involves use both of the complete CSD and its associated software systems, and of a specially chosen teaching subset of 500 CSD entries which can be freely examined using the new CSD web interface *WebCSD* (Thomas *et al.*, 2010 ▶). As part of the initiative, we have created a set of undergraduate teaching examples, available through the CCDC website (CCDC, 2010*a* ▶), which will be discussed below. We have also become involved in the US Biannual Conferences on Chemical Education, and a symposium at the Fall 2009 meeting of the American Chemical Society (Division of Chemical Education) specifically addressed the potential of the CSD in chemical education. Speakers at this symposium have kindly made their presentations freely available *via* the CCDC website (CCDC, 2009 ▶). Some of these presentations are referenced individually in this paper under the name of the presenting author.

## 2. Data sources and software

A number of CCDC teaching resources are available free of charge to the educational community:

(*a*) A teaching subset of *ca* 500 CSD entries illustrating a wide range of three-dimensional structural issues.

(*b*) A web-based interface for browsing the teaching subset.

(*c*) A downloadable version of the CCDC’s *Mercury* (Macrae *et al.*, 2006 ▶) visualizer.

(*d*) Example teaching exercises that utilize the teaching subset.

The software and database entries are exactly as used by researchers in the field, *i.e.* they are not specially ‘reduced’ versions for classroom use.

However, more advanced teaching applications require access to the complete CSD System, and this is supplied to individual institutions for a small cost-recovery fee which is further reduced for non-PhD-awarding institutions. The full CSD System includes a suite of computer programs, described in §[Sec sec2.1]2.1, that facilitate search, analysis and visualization of CSD information. Additionally, online access to the database *via WebCSD* (Thomas *et al.*, 2010 ▶; see §[Sec sec2.2]2.2) is available to those institutions with an unlimited site-wide CSD System licence.

### 2.1. The CSD System

The CSD System includes the following elements:

(i) The Cambridge Structural Database. Compilation of the CSD began in 1965, and the information content of a structural entry is fully described elsewhere (Allen, 2002 ▶); only a brief summary is presented here. The CSD records data from single-crystal X-ray and neutron diffraction studies of C-containing compounds: organics and metal–organics. Powder structures are also included. Each entry, identified by a CSD refcode, records the primary numerical results of the analysis: three-dimensional atomic coordinates, cell dimensions and space group, together with (*a*) a formal two-dimensional chemical diagram and searchable chemical connection table, (*b*) full bibliographic reference, including the DOI, (*c*) chemical name and formula, and (*d*) other information that may be present in the published or directly deposited report.

(ii) *ConQuest* (Bruno *et al.*, 2002 ▶) permits searches of all CSD information fields, and most importantly the location of chemical substructures and intermolecular interactions defined using both two-dimensional connectivity and three-dimensional geometric constraints. Apart from locating the required crystal structures, *ConQuest* also outputs user-defined geometrical parameters for the structure or substructure of interest.

(iii) *Vista* allows the geometrical data retrieved from the CSD to be presented as a spreadsheet, and displayed as histograms and scattergrams using Cartesian or polar axes. *Vista* will also perform a variety of statistical analyses on the retrieved data, *e.g.* regression, principal components analysis *etc*., and is important in the derivation of mean geometry and in the identification of conformational preferences in chemical substructures. Other software, *e.g.* Microsoft *Excel*, can also read *ConQuest* output and can be used in data analysis.

(iv) *Mercury* (Macrae *et al.*, 2006 ▶) provides both basic and advanced functionality for viewing molecules in three dimensions, and facilitates the exploration and analysis of extended crystal structure motifs and packing arrangements. Molecules may be displayed in a variety of styles and colouring schemes. The contents of any number of unit cells (or fractions of unit cells) can be displayed and crystal structures may be viewed along direct or reciprocal cell axes, or perpendicular to any specified atomic plane. The display of space-group symmetry elements is also facilitated (see §[Sec sec5]5). Distances, angles and torsions can be measured, and items such as atom labels and unit-cell axes can be shown. Coupled with this general functionality is the ability to build and explore extended networks of molecules and their linking contacts such as hydrogen bonds. This interactive exploration of crystal structures can greatly assist the student’s comprehension of the importance of hydrogen bonds, by studying the extended packing arrangement of the structure, and the key intermolecular interactions involved in molecular aggregation.

(v) Knowledge bases. *Mogul*, a knowledge base of intramolecular geometry (Bruno *et al.*, 2004 ▶), and *IsoStar*, a knowledge base of intermolecular interactions (Bruno *et al.*, 1997 ▶), are also part of the distributed CSD System and provide rapid access to CSD information. These knowledge bases allow students to answer questions such as ‘what is the preferred solid-state conformation of *n*-butane?’ or ‘is the carbonyl or the ether O atom of an ester group more likely to form a hydrogen bond?’ but without the need to construct complex search queries.

### 2.2. WebCSD

The CSD has recently been made internet-accessible through *WebCSD* (Thomas *et al.*, 2010 ▶): a web-based search engine for interrogating the CSD. *WebCSD* allows institutions with site-wide CSD System access to search the full database of over 500 000 structures from any computer at their site using just a standard web browser, and without the need for any local software installations. This ease of access makes the online version of the CSD ideal for use in classroom and computational teaching laboratory environments. In addition to full text and numeric searching, *WebCSD* allows two-dimensional chemical substructure queries to be defined using an embedded sketcher – thus allowing rapid retrieval of structures of interest. Additionally, a complementary two-dimensional structure-based search option will locate those CSD entries having the highest molecular similarity to a drawn query molecule. This allows non-expert users to locate specific structures of interest, and their analogues, without having to learn the subtleties of substructure searching.

**Figure 1:**
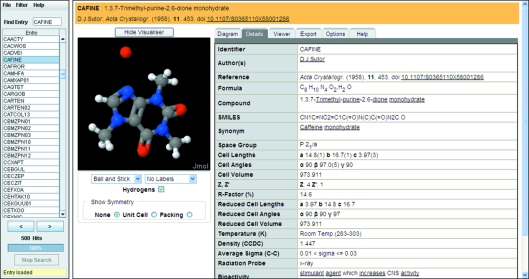
*WebCSD* interface showing chemical information for caffeine (teaching subset entry CAFINE).

The crystal structure information accessed either through searches or by simply browsing the CSD is easily accessible in a single pane (Fig. 1 ▶). This includes the two-dimensional chemical diagram, full bibliographic information, including author names and journal reference (with links to the original publication), and other text and numerical data, for example, compound name, molecular formula, precision indicators *etc*. However, crystal structure information is inherently focused on three-dimensional data and *WebCSD* provides a choice of two different three-dimensional viewers as embedded Java applets: *Jmol* (2010 ▶) or *OpenAstexViewer* (2010 ▶). These molecular viewers provide a range of display styles as well as atom labelling and tools to measure distances, angles and torsions. *Jmol* (the default viewer in *WebCSD*) also supports some crystallographic viewing options such as the display of a full unit cell or a packing range of 3 × 3 × 3 unit cells.

The embedded viewer options allow *WebCSD* to be used effectively without the need for additional client-side applications. However, crystal structures can also be exported from *WebCSD* into *Mercury* (either a single structure as a CIF, or many as a list of CSD refcodes) for more advanced structure visualization and analysis. Thus *WebCSD* can act as a springboard for more advanced studies – allowing very fast searches with links to desktop applications for further investigation of the results.

### 2.3. The CSD teaching subset

A subset of *ca* 500 structures that have important chemical education applications have been carefully selected from the full CSD of over half a million entries. These structures are available to the educational community free of charge and can be accessed *via* either

(*a*) a freely accessible online version of the *WebCSD* interface (*WebCSD*, 2010 ▶), or

(*b*) a downloadable version of the CCDC’s *Mercury* visualizer (CCDC, 2010*c* ▶).

Table 1 ▶ contains statistics for selected structure types in the teaching subset. The composition of the 500-structure teaching subset is described more fully elsewhere (Battle *et al.*, 2010*a* ▶). While the subset has been selected specifically for the teaching content of the structures included, the CSD comparison statistics included in Table 1 ▶ show that the subset is reasonably representative of the CSD as a whole.

Many of the key molecules used in standard chemical texts to exemplify core concepts and principles in the undergraduate chemistry curriculum are included in the teaching subset. These include, *inter alia*, (*a*) compounds used to illustrate fundamental concepts of bonding and structure, *e.g.* benzene (CSD refcode BENZEN02), diborane (GAFLAA) and ferrocene (FEROCE27); (*b*) compounds used to exemplify conformational issues, including a wide variety of ring systems, *e.g.* the equi-energetic half-chair and envelope conformations of cyclopentane (LISLOO and IHIPOE, respectively), and the energetically preferred chair form of cyclohexane (CYCHEX); and (*c*) compounds commonly used to teach stereochemistry, *e.g.* the l-(*R*)- and d-(*S*) forms of alanine (LALNIN23 and ALUCAL05, respectively), and the three stereoisomers of tartaric acid [the two enantiomers (TARTAC and TARTAL04) and the achiral *meso* form (TARTAM)]. In addition to these key molecules it is important that the teaching subset accurately represents the massive chemical and structural diversity within the CSD. Thus, many major functional groups are represented, as are a wide range of broader chemical classes, including carbohydrates, nucleotides, amino acids, steroids, porphyrins, alkaloids, organometallics, metal complexes, *catena* structures and high polymers. A diverse range of molecular geometries are also represented, including simple examples (composed entirely of main-group elements) of all the main VSEPR structure types (see, *e.g.*, Housecroft & Sharpe, 2005 ▶). Furthermore, the subset includes examples of 80 different crystallographic space groups. A broad range of molecules that can be used to demonstrate concepts of molecular symmetry are also included.

Structure quality was another important consideration. In order to preserve the unique challenges and advantages afforded by real measured data, no structures were modified in any way before inclusion. Therefore the teaching subset contains, for example, a small number of disordered structures (5.8%). Also, some structures have been determined more than once, and wherever possible the ‘best’ determination of a particular structure, according to the definitions of van de Streek (2006 ▶), was selected for inclusion in the subset.

## 3. Teaching applications using the CSD subset *via WebCSD*

### 3.1. Teaching modules

It is obvious that individual structures, or small groups of structures, from the CSD teaching subset can be used to great effect in illustrating three-dimensional chemical concepts – conformation, stereochemistry, chirality, hydrogen bonding, metal coordination geometry *etc.* – in any teaching environment. However, the subset can be used creatively to construct teaching modules that involve the student in a fully interactive learning experience. The CCDC website (CCDC, 2010 ▶) has five teaching modules (Table 2 ▶) based entirely on use of the teaching subset and *WebCSD*. Four more modules that require the full CSD System (Table 3 ▶) are discussed in §[Sec sec4]4. Each teaching module comprises the following components: (*a*) chemical background to the module, (*b*) the objectives of the exercise, (*c*) database and software requirements, (*d*) steps required to complete the module, fully illustrated by screen shots, and (*e*) a summary of the key concepts that have been learned. The topics and objectives of these modules, together with an overview of the student’s interaction with the system, are summarized in Tables 2 ▶ and 3 ▶. These modules are fully discussed elsewhere (Battle *et al.*, 2010*b* ▶), so here we briefly exemplify their value in the teaching of (*a*) organic and (*b*) inorganic chemistry with an expanded description of one module in each of these chemical categories.

While we are confident that these exercises are of sound pedagogical value, a formal assessment of the learning efficacy of these specific modules has yet to be carried out. Rather, our core purpose here has been threefold: (*a*) to illustrate how the CSD teaching subset can be used in teaching, (*b*) to encourage others to suggest additional structures for inclusion in the subset (email teaching@ccdc.cam.ac.uk) and to derive or suggest additional examples for inclusion in the teaching section of the CCDC website, and (*c*) to encourage others to assess their student learning outcomes upon adoption of these tools in order to help provide a more authoritative discussion of their pedagogical value.

### 3.2. Organic chemistry

**Figure 2:**
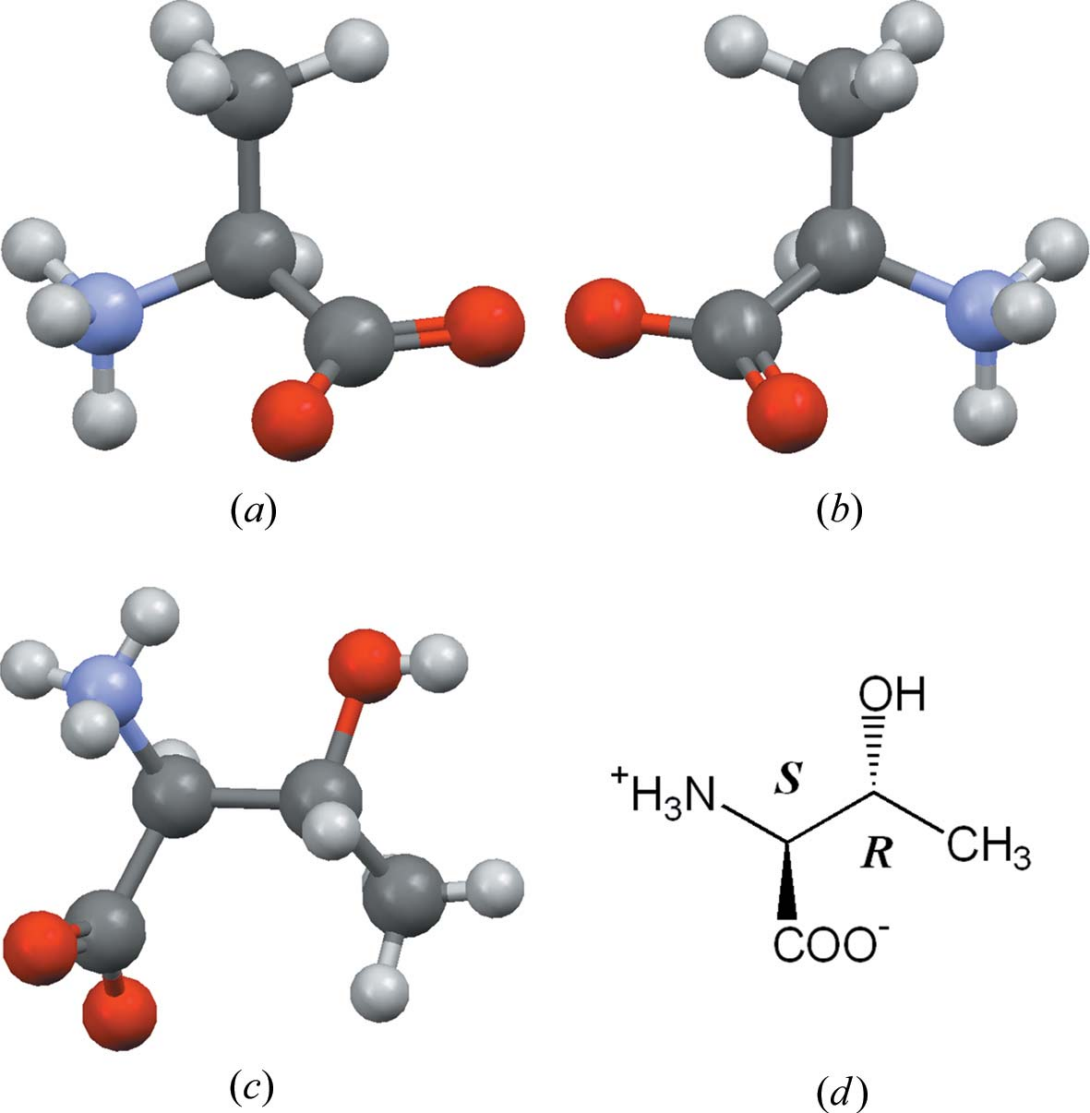
(*a*) l-(*R*)-alanine (LALNIN23), (*b*) d-(*S*)-alanine (ALUCAL05), (*c*) (2*S*,3*R*)-threonine (LTHREO01) and (*d*) (2*S*,3*R*)-threonine (two-dimensional wedge/dot bonds representation).

Module 3 on stereochemistry and chirality introduces the importance of these concepts in organic chemistry, biological systems and drug action. The value of experimental three-dimensional structural information in illustrating basic concepts is reinforced. The student is first asked to compare two crystal structures of alanine: natural l-(*R*)-alanine (LALNIN23) and the d-(*S*) form (ALUCAL05). These two structures are mirror images and cannot be superimposed. Various manipulations of the structures are performed using *WebCSD*, including viewing each along the C—H bond (Figs. 2 ▶ *a* and 2 ▶ *b*) and comparing the results. From this, the tutorial develops rules for chirality perception, and students are asked to examine a number of structures and determine if they contain a stereogenic centre or not. Next, the concept of *R* and *S* enantiomers is introduced by defining the priority ordering of substituents at the chiral C atom in alanine. Students are asked to make *R* or *S* assignments for several other CSD structures, including carvone (RERXIV), adrenaline (ADRENL) and ibuprofen (JEKNOC10).

The tutorial then considers compounds having more than one stereogenic centre, through an examination of threonine (LTHREO01; Figs. 2 ▶ *c* and 2 ▶ *d*) which has two chiral centres identifiable as (2*S*,3*R*). The student is asked to determine which other stereoisomers can exist for threonine (four) and to determine their relationship as two pairs of mirror-image (enantiomeric) structures: (*a*) (2*R*,3*R*)/(2*S*,3*S*) and (*b*) (2*R*,3*S*)/(2*S*,3*R*). The diasteromeric relationship between non-mirror-image pairs is then introduced and exemplified *via* a study of ephedrine (EPHEDR01) and pseudoephedrine (PSEPED01). The final segment of the tutorial discusses the stereoisomers of tartaric acid (TARTAC, TARTAL04 and TARTAM), where the two stereogenic centres might be expected to generate four stereoisomers, paired up as for threonine above. In fact, we see only three stereoisomers: the mirror-image optically active forms (2*S*,3*S*) (TARTAC) and (2*R*,3*R*) (TARTAL04), and TARTAM which is both (2*R*,3*S*) and (2*S*,3*R*) since the molecule has a mirror plane bisecting the central C—C bond, so that no absolute distinction can be made between C_2_ and C_3_. Such compounds are not optically active (achiral) and are termed *meso* compounds.

The chirality tutorial is followed by suggestions for more advanced exercises covering other kinds of molecules that can display chirality, *e.g.* (*a*) compounds with other quadrivalent atoms; (*b*) compounds with tervalent chiral atoms, *e.g.* pyramidal N in which the lone pair acts as the fourth substituent; (*c*) compounds that exhibit molecular chirality; (*d*) chirality due to restricted rotation, where a tetra-*ortho*-substituted biphenyl is provided as an example for study; and (*e*) chirality due to helicity, as exemplified by the hexahelicenes, which illustrate how clockwise and counterclockwise helices are not superimposable.

### 3.3. Inorganic chemistry

Module 4 (Table 2 ▶) illustrates the VSEPR method. The basic shapes of molecules tend to be controlled by the number of electrons in the valence shell of the central atom. The valence-shell electron-pair repulsion (VSEPR) model facilitates the prediction of three-dimensional molecular shapes. The tutorial begins by asking students to predict three-dimensional structures for di-, tri- and tetrachloromercury, to compare their predictions with structures in the CSD teaching subset – OKAJOZ (linear HgCl_2_), KUSMAM (trigonal planar HgCl_3_^−^) and KEYZUK (tetrahedral HgCl_4_^2−^) – and to confirm these shapes by measuring Cl—Hg—Cl angles. This agrees with the VSEPR model, which predicts that preferred shapes will ensure that regions of enhanced electron density will take up positions as far apart as possible to generate a minimum-energy arrangement. A table is then provided of the ideal VSEPR geometries for compounds containing from two to eight electron pairs. Using [PF_6_]^−^ as an example, students are asked to determine the number of electron pairs present (six), predict the preferred three-dimensional shape (octahedral), and confirm this by examining and measuring valence angles in WINFAA. Several other CSD examples of three–six coordination are then provided to be studied in the same way.

**Figure 3:**
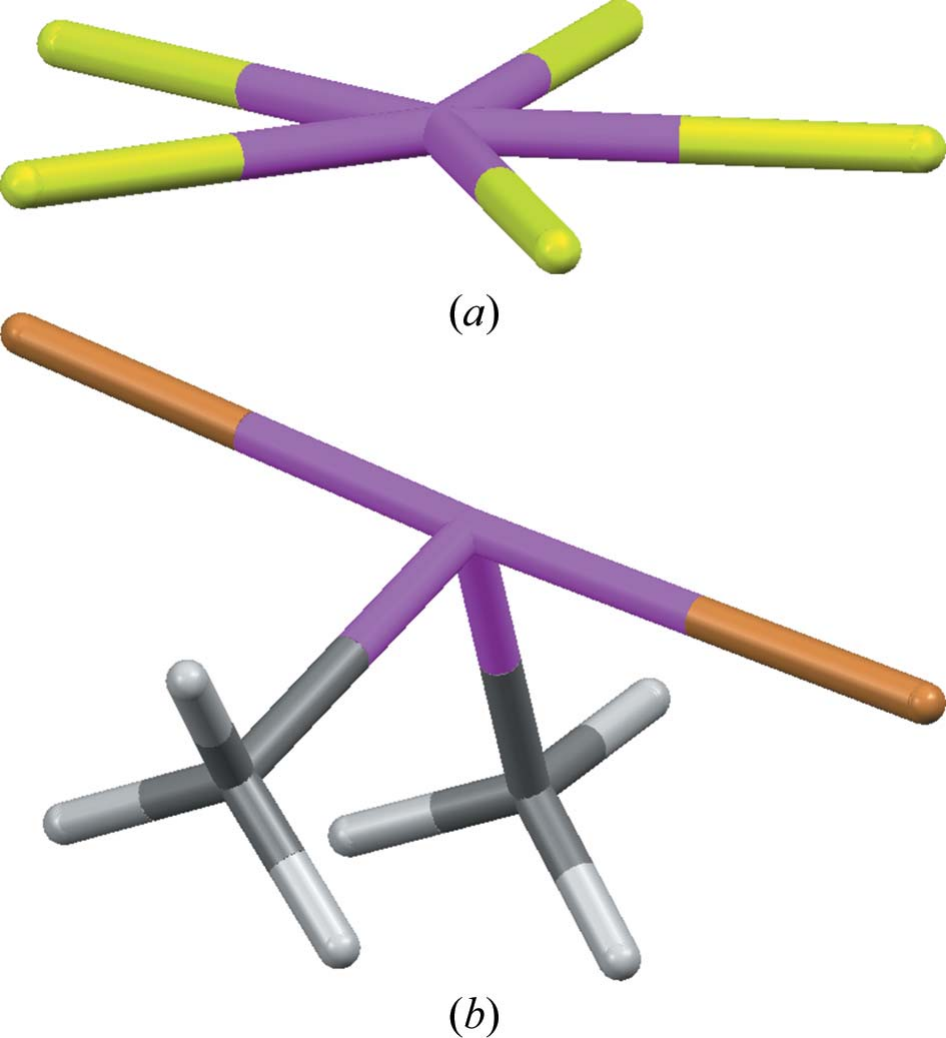
(*a*) The planar [XeF_5_]^−^ ion (present in CSD structure SOBWAH) maximizes the lone-pair repulsion so that the F atoms occupy the apices of a pentagonal bipyramid. (*b*) The ‘seesaw’ shape of dibromodimethylselenium (RIZMIW), where the lone pair occupies an equatorial position in a trigonal bipyramid to minimize lone-pair–bonding-pair repulsions.

The tutorial then considers the effect of lone pairs, using the [XeF_5_]^−^ ion present in SOBWAH (Fig. 3 ▶ *a*), which shows the ion to be a planar five-coordinate species – why? The tutorial provides the answer in terms of minimizing lone-pair–lone-pair repulsions. The student is then asked to rationalize (*a*) the ‘seesaw’ shape of dibromodimethylselenium (RIZMIW; Fig. 3 ▶ *b*), where the lone pair occupies an equatorial position in a trigonal bipyramid to minimize lone-pair–bonding-pair repulsions, and (*b*) the three-dimensional structure of the water solvent in MUSIMO01, which has an H—O—H angle less than the normal tetrahedral value. The tutorial concludes by suggesting a further dozen compounds for application of the VSEPR method, together with CSD refcodes for confirming the predictions.

### 3.4. Hydrogen bonding

**Figure 4:**
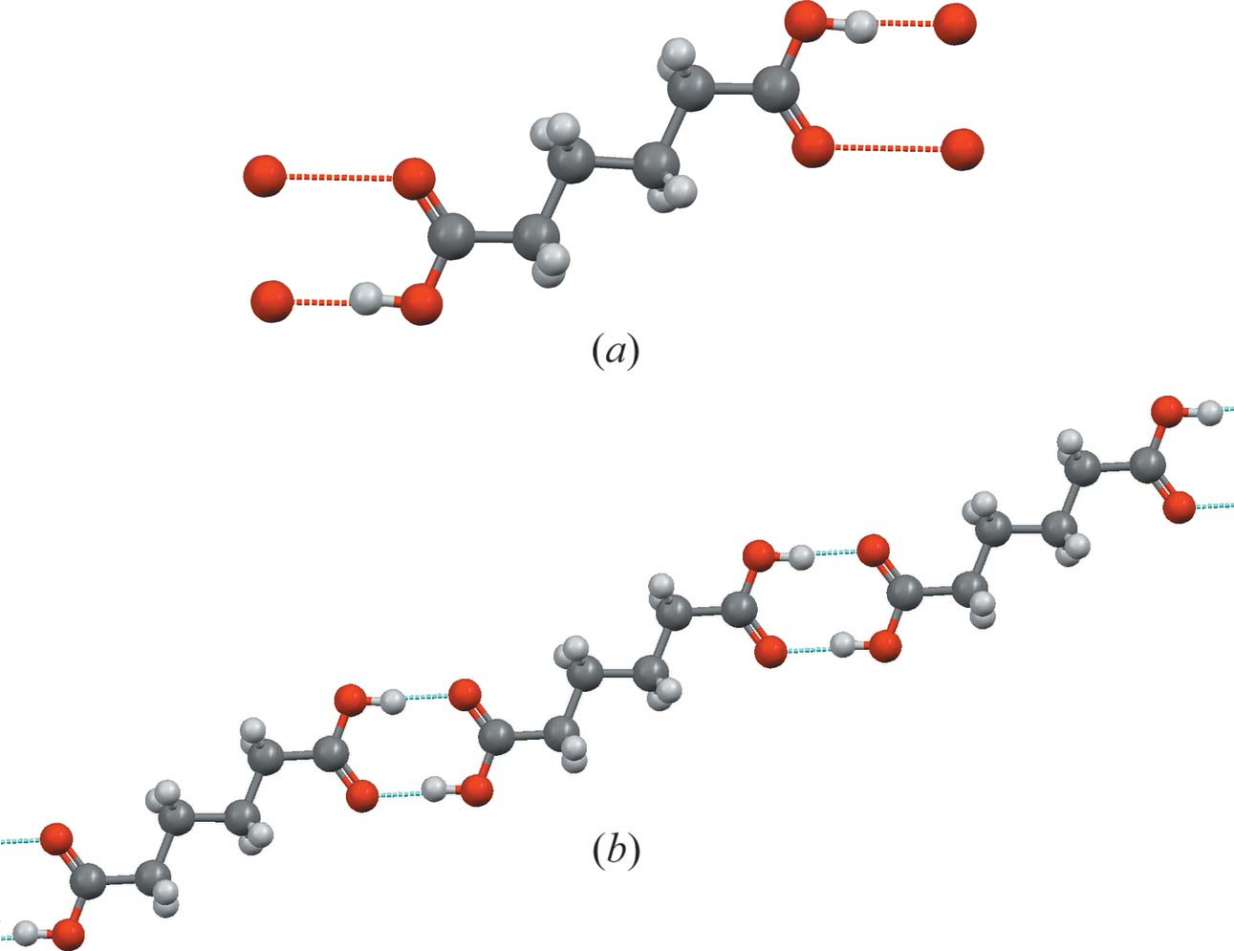
*Mercury* plots exploring hydrogen bonding in the CSD teaching subset. (*a*) Adipic acid (ADIPAC04) with ‘hanging’ hydrogen-bonded O—H⋯O contacts in red, and (*b*) extended chain of molecules formed by carboxylic acid dimers obtained by clicking on the hanging contacts in part (*a*).

The basic concepts of hydrogen bonding as an electrostatic donor (*D*)–acceptor (*A*) interaction of the form *D*—H^δ+^⋯*A* ^δ−^ that is responsible for the formation of many extended structures, and which is vital in biological systems, is readily illustrated by use of structures from the teaching subset. For maximum effectiveness, these should be downloaded and viewed with the *Mercury* software (Macrae *et al.*, 2006 ▶), which detects hydrogen bonds according to (configurable) geometrical criteria, and shows these as ‘hanging’ (red) contacts from the target molecule (*e.g.* as for adipic acid, ADIPAC04; Fig. 4 ▶ *a*); clicking on these hanging contacts then expands the molecular array, forming a chain of carboxylic acid dimers (Fig. 4 ▶ *b*). Students might then be asked to examine hydrogen bonding in other acids, *e.g.* acetic acid (ACETAC07), benzoic acid (BENZAC02), acrylic acid (ACRLAC02) *etc*. They should note that acetic acid is unusual in forming an extended *catena* structure, rather than the cyclic dimer exhibited in Fig. 4 ▶(*b*). Further insights can be gained by inspecting the hydrogen bonding exhibited by other functional groups, such as amide, hydroxy *etc*. Students might then be asked to examine the more complex hydrogen-bonding possibilities available in simple amino acids, such as the d- and l-alanines (ALUCAL05, LALNIN23), which are zwitterionic and have three N—H donors and two O=C acceptors; they might extend this study to other amino acids, such as l-serine (LSERIN01), which has an additional OH donor/acceptor, and l-cystine (LCYSTI10) with six N—H donors and four O=C acceptors. Studies of this type provide insights into the hydrogen-bonding complexities that exist in peptides and in protein structures.

## 4. Teaching applications using the full CSD System

While the CSD teaching subset and its application modules provide a significant resource for chemical educators, there are many cases where the full CSD System is essential to make an educational point (Tables 2 ▶ and 3 ▶). This is particularly true when introducing students to variance in real experimental observations, or in examples where many hundreds of observations are required to generate statistically meaningful trends from the structural data. In this section, we trace some important themes in modern organic and inorganic structural chemistry from an educational viewpoint. Some of the examples derive directly from published research applications of the CSD.

### 4.1. Organic chemistry

#### 4.1.1. Mean molecular dimensions

The derivation of mean molecular and intermolecular geometrical parameters has been a major research use of the CSD. In the late 1980s, the CCDC and collaborators at the University of Bristol, UK, published printed compilations of mean bond lengths in organic molecules (Allen *et al.*, 1987 ▶) and in organometallics and complexes of the *d*- and *f*-block metals (Orpen *et al.*, 1989 ▶). These compilations in themselves provide key information for students, but it is also informative for students to appreciate the data retrieval and analysis methods that were used in the generation of the mean values given in the tables. A simple example concerning the mean Sb—F distance in SbF_6_ ions can be accessed *via* the CCDC website (CCDC, 2010*a* ▶) (see Table 3 ▶), but we illustrate the fundamentals by expanding on Module 2 of Table 2 ▶ – ring strain and conformation – by (*a*) evaluating the mean C—C bond length in an unstrained C—C single bond, (*b*) comparing this value with mean C—C bond lengths in the strained carbocycles cyclopropane and cyclobutane, and (*c*) performing a more detailed analysis of ring buckling in four-membered carbocycles.

**Figure 5:**
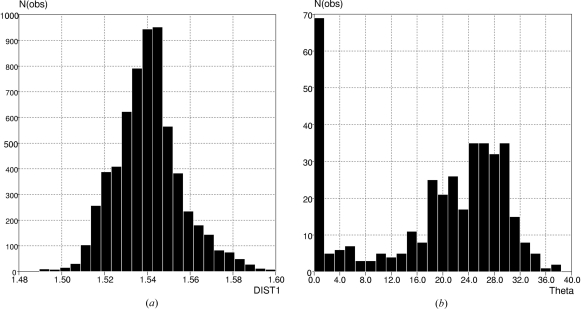
CSD plots of (*a*) mean bond length for central C—C bond in acyclic (C*sp* ^3^)_2_—CH—CH—(C*sp* ^3^)_2_ substructures, and (*b*) angle of pucker (θ) in nonfused/nonbridged cyclobutane rings.

Since there are millions of C—C bonds in the CSD, we restrict our *ConQuest* search for unstrained examples to the specific substructure (C*sp* ^3^)_2_—CH—CH—(C*sp* ^3^)_2_. In order to avoid C—C bonds from strained rings, *ConQuest* can be instructed to select only acyclic central bonds, and to avoid hits that contain any additional direct links between atoms specified in the query substructure. After removal of 33 obvious outliers, the histogram of Fig. 5 ▶(*a*) was obtained, giving a mean central C—C bond length of 1.540 Å, with a sample s.u., σ_s_, of 0.016 Å, and an s.u. of the mean, σ_m_, of <0.001 for the 6301 observations. Similar CSD searches were carried out for cyclopropane and cyclobutane rings, using *ConQuest* settings to avoid fusion to any other ring, with the results for mean C—C bond lengths set out in Table 4 ▶. For cyclobutane, the dihedral angle about one of the ring diagonals (θ) was also calculated for each ring, and the θ distribution is shown in Fig. 5 ▶(*b*). This shows that the majority of rings are puckered, with a preference for θ values in the range 15–35°. The puckering relieves the strain in the ring due to the short 1,3-(C,C) distances and the perfectly eclipsed C—*X* substituents that occur in the planar form. Nevertheless, 69 of the 383 rings in this sample are perfectly planar in crystal structures, usually occurring around a centre of symmetry. The increased strain in these planar rings is reflected in the data of Table 4 ▶, which show that the mean C—C bond in planar cyclobutane is longer by almost 0.02 Å than the C—C bond in puckered rings. Given that cyclopropane is planar with fully eclipsed C—*X* substituents, students might imagine that the mean C—C distance here would also be longer than for an unstrained C—C bond. Students will see, however, that the mean C—C bond in cyclopropane is in fact very much shorter than any of the other C—C bonds quoted in Table 4 ▶. This leads immediately to a discussion of the ‘banana’ bonds and Walsh orbitals that explain bonding in cyclopropane (Walsh, 1949 ▶; Jorgensen & Salem, 1973 ▶), and to a discussion of the ethylenic nature of the cyclopropane ring (Charton, 1970 ▶) in organic systems.

#### 4.1.2. Conformational analysis and stereochemistry

**Figure 6:**
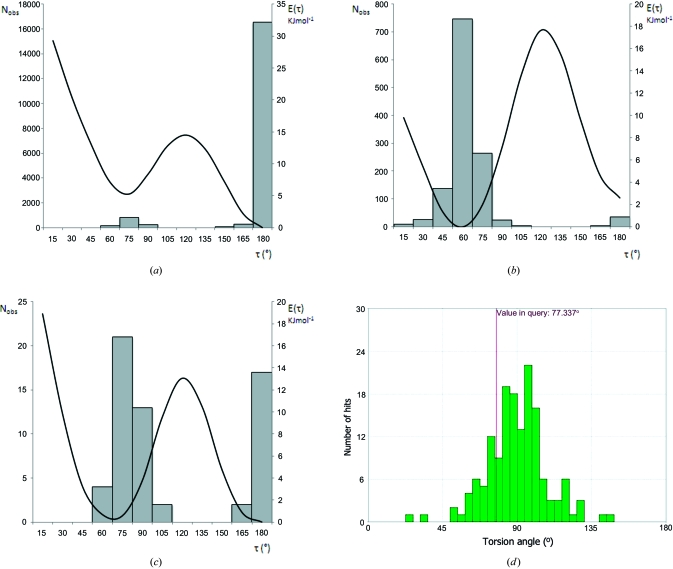
Torsional distributions from the CSD about the central C—C bond in three simple substructures: (*a*) butane (CH_3_—CH_2_—CH_2_—CH_3_), (*b*) 2-methylbutane [(CH_3_)_2_—CH_2_—CH_2_—CH_3_] and (*c*) 2,3-dimethylbutane [(CH_3_)_2_—CH_2_—CH_2_—(CH_3_)_2_]. Potential energy curves calculated using *Chem3D Ultra* (CambridgeSoft, 2009 ▶) are superimposed on the distributions. (*d*) The distribution of C_ar_—C_ar_—S—C torsion angles in arylsulfones from the CCDC’s *Mogul* knowledge base (2008 release).

The usual student introduction to conformational analysis is the relationship between potential energy (*E*) and the H—C—C—H torsion angle (τ) in ethane: the equi-energetic staggered conformations (illustrated in the CSD teaching subset by ETHANE01) with τ = ±60 (±*gauche*) and 180° (*anti*) are favoured over the fully eclipsed conformers (τ = 0 and ±120°) by around 12 kJ mol^−1^ (Eliel & Wilen, 1994 ▶). However, as the H atoms in ethane are progressively replaced by larger groups, *e.g.* methyl groups as in butane (Fig. 6 ▶ *a*), 2-methylbutane (Fig. 6 ▶ *b*) and 2,3-dimethylbutane (Fig. 6 ▶ *c*), then the *gauche* and *anti* forms cease to be equi-energetic and the proportion of *gauche*:*anti* conformers varies considerably, as shown by the CSD τ distributions presented in Fig. 6 ▶. These distributions have been generated from the November 2009 CSD release using the search and retrieval criteria described by Allen *et al.* (1996 ▶) and clearly reflect the changes in the *gauche*:*anti* energy relationship shown in the potential energy curves that are superimposed on the torsional distributions in Fig. 6 ▶. The energy curves have been calculated using *Chem3D Ultra* (CambridgeSoft, 2009 ▶) and an MM2 force field, software that is likely to be available in a teaching environment. Allen *et al.* (1996 ▶) show similar *E*–τ diagrams for a further nine substructures having freely rotatable acyclic C—C, C—O and C—S bonds that also have educational value. Crystal structure data also provide excellent and simple examples of ring conformations and stereochemical features for use in teaching. Parent cyclohexane (CSD teaching subset: CYCHEX) illustrates the archetypal chair-form six-membered ring, while the many examples of α- and β-pyranose sugars in the main CSD provide valuable insights into axial and equatorial stereochemistry and diastereoisomerism.

Students should be made aware that conformational knowledge obtained from crystal structures is widely used in the design of novel molecules, particularly in the discovery of novel pharmaceuticals, and a recent review by Brameld *et al.* (2008 ▶) covers this topic in a highly accessible manner. These authors stress the importance of the massive chemical diversity of the CSD and of the huge reservoir of conformational information that is available in the *Mogul* knowledge base (Bruno *et al.*, 2004 ▶) at the click of a few buttons in its interface. Fig. 6 ▶(*d*) shows the distribution of C_ar_—C_ar_—S—C torsions in arylsulfones generated using the *Mogul* interface.

#### 4.1.3. Hydrogen bonding and other intermolecular interactions

**Figure 7:**
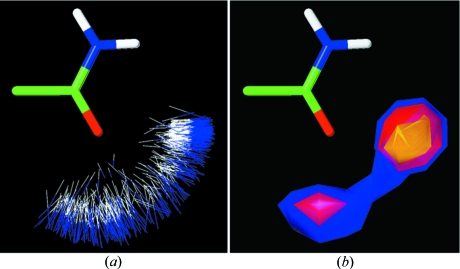
*IsoStar* scatterplots of an N—H contact group around an amide central group: (*a*) standard plot and (*b*) contoured plot.

It is necessary to use the full CSD System in order to obtain a complete overview of the spatial and geometric characteristics of hydrogen-bonded systems and of intermolecular interactions not mediated by hydrogen. The *IsoStar* knowledge base (Bruno *et al.*, 1997 ▶) is particularly valuable in visualizing interactions between functional groups, defined as central groups and contact groups. The library contains more than 25 000 interaction scatterplots derived from the CSD (20 000 plots), with the remainder coming from higher-resolution (better than 2 Å) protein–ligand complexes from the PDB (Berman *et al.*, 2000 ▶). *IsoStar* also presents theoretical energy minima for >1500 key interactions. A typical *IsoStar* plot for an N—H contact group and an amide central group is shown in its ‘native’ and contoured forms in Figs. 7 ▶(*a*) and 7 ▶(*b*), and shows the preference for the N—H donor to interact with the lone pairs of the amide oxygen. Since the *IsoStar* library contains information for 300 central groups and 48 contact groups, it represents a mine of information not just for researchers but also for students at many levels of instruction. The ability of the *ConQuest* program to search for specified intermolecular interactions is fully illustrated elsewhere (see, *e.g.* Allen *et al.*, 2010 ▶; Allen & Motherwell, 2002 ▶) and allows students to quantify, *e.g.*, intermolecular hydrogen bonds (*D*—H⋯*A*—*X*) in terms of (*a*) their *D*⋯*A* and H⋯*A* distances, (*b*) the angle of hydrogen directionality (*D*—H⋯*A*), and (*c*) the angle of hydrogen approach to the acceptor (H⋯*A*—*X*) to examine possible lone-pair involvement in the interaction. Comparisons of hydrogen-bonded distances can also give insights into the relative strengths of interactions involving different functional groups, but computational chemistry procedures are better suited to this task and would form a useful extension of the database studies in the student curriculum.

#### 4.1.4. Reaction pathways

**Figure 8:**
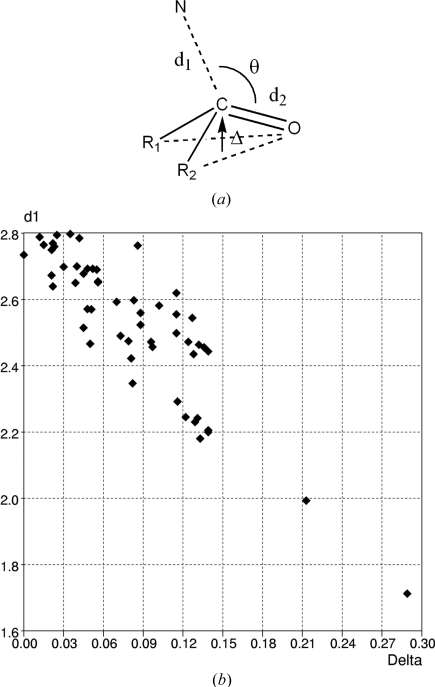
Analysis reaction pathways using CSD data: attack of a nitrogen nucleophile on a carbonyl centre (after Bürgi & Dunitz, 1986 ▶). (*a*) Substructure search fragment and geometrical parameters used to locate and plot the reaction pathway, and (*b*) plot of the nonbonded N⋯C distance (*d*_1_) *versus* the increasing pyramidality at the carbonyl C atom, measured by the parameter Δ in part (*a*).

One of the earliest and most significant correlations of crystal structure information with chemical activity was the study of reaction pathways (Bürgi & Dunitz, 1986 ▶, 1994 ▶), particularly the use of short N⋯C=O contacts to map the attack of a nitrogen nucleophile on a carbonyl centre as illustrated in Fig. 8 ▶(*a*). The original analysis used just six N⋯C distances ranging from 2.91 Å (nonbonded) to fully bonded N—C values at 1.49 Å, and including N⋯C values of 2.58, 2.55, 1.88 and 1.64 Å to complete the range. These authors used the geometrical construct of Fig. 8 ▶(*a*) to map and correlate the available data at a time when <15 000 structures were recorded in the CSD. A recent CSD search located 32 examples of the fragment of Fig. 8 ▶(*a*) having a nonbonded N⋯C distance (*d*_1_) < 2.6 Å. A plot of *d*_1_ *versus* the C pyramidality, Δ, is essentially linear (Fig. 8 ▶ *b*): as the N nucleophile approaches the carbonyl C atom, the carbonyl group deviates increasingly from planarity and the length of the C=O bond also increases, *i.e.* the C atom is in the early stages of changing its hybridization from *sp* ^2^ to *sp* ^3^. Importantly also, the angle of nucleophilic approach, N⋯C=O, is always larger than the 90° that might be expected and is also reasonably constant: the mean value for the 32 fragments in this analysis is 107 (2)°. This result can be related to changes that occur in the molecular orbitals as the reaction proceeds, and this nucleophilic approach route has become known as the Bürgi–Dunitz trajectory, a topic that is now included in many undergraduate organic chemistry texts (see, *e.g.*, Clayden *et al.*, 2000 ▶). A number of chapters in the two-volume book *Structure Correlation* (Bürgi & Dunitz, 1994 ▶), particularly those by Cieplak (1994 ▶), Burgi & Shklover (1994 ▶) and Auf der Heyde (1994 ▶), suggest many other examples of structure–reactivity correlations that are suitable for teaching purposes, while Wheeler (2009 ▶) has described a teaching module that conceptualizes reaction mechanisms using crystallographic data, and which is delivered to undergraduate chemists at the University of Eastern Illinois. In a recent paper in the chemical education literature, Wackerly *et al.* (2009 ▶) also use structure correlation principles and the CSD to examine the geometry at N and P atoms that are bonded to three C atoms, and to correlate bond lengths, twist angles and pyramidalization in *N*,*N*-disubstituted anilines as a learning exercise.

### 4.2. Inorganic chemistry

While organic chemists have developed the wedge/dot bond system for depicting pseudo-three-dimensional representations for the compounds (principally) of carbon, nitrogen and oxygen, the three-dimensional nature of inorganic compounds is much more complex, with metal coordination numbers greater than four being commonplace. For this reason, visualizations of crystallographically determined molecular structures are a pre-requisite to understanding and are commonplace in undergraduate inorganic chemistry texts. This dates back to the earliest texts (*e.g.* Cotton & Wilkinson, 1980 ▶, and earlier editions) that chart the renaissance of the subject in the 1960s, and is continued in more modern texts, such as Housecroft & Sharpe (2005 ▶) who show more than 250 three-dimensional structures of key molecules and ions, most of which occur in the CSD. Examples in their book range from the common sulfur allotrope, S_8_ (FURHUV), to the rather complex magnetic resonance imaging contrast agent aqua[diethyl­enetriamine-bis(acetic acid methylamide)­triacetato]gadolinium, [Gd(DTPA-BMA)(H_2_O)] (trade name Omniscan; UDOMOP as hexahydrate). This long-term use of crystal structure information to teach inorganic chemistry is a manifestation of a synergistic relationship: crystal structure analysis is often the only analytical method suitable for characterizing novel inorganic compounds, and it is natural for inorganic chemists to use these images in their teaching activities. As evidence of the inorganic chemistry–crystallography synergy, Table 1 ▶ shows that 53.1% of the compounds in the CSD contain a transition metal, and a further 6.3% contain a main-group metal. With nearly 300 000 metal-containing structures, the CSD obviously contains a plethora of information of value in chemical education. Thus, the database may be used to illustrate coordination stereochemistry by viewing the structures of the *cis* (CCPYPT) and *trans* (CLPYPT) isomers of Cl_2_(py)_2_Pt, or the *cis* (HELREV) and *trans*,*trans*,*trans* (HOKCUF) isomers of Ru(Cl)_2_(CO)_2_(PPh_3_)_2_, or perhaps by comparing [(+)(en)_3_Co]^3+^ with [(−)(en)_3_Cr]^3+^ in the same structure (COENCL). We now briefly summarize some specific examples where the full CSD System has enormous value in the teaching of inorganic chemistry.

#### 4.2.1. Jahn–Teller distortions in octahedral Cu^II^ complexes

**Figure 9:**
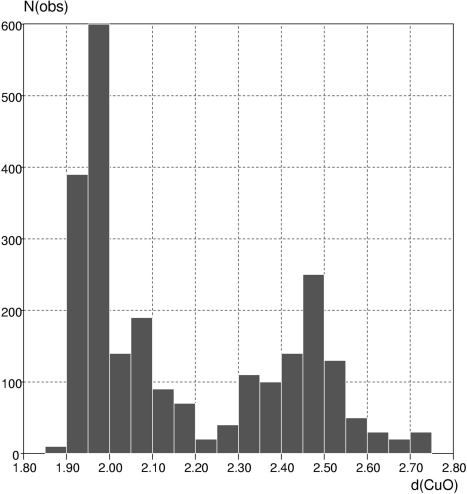
The Jahn–Teller effect illustrated by a histogram of Cu—O distance in CuO_6_ substructures.

There are over 600 examples of [*M*(H_2_O)_6_]^*n*+^ complex ions in the CSD, including examples containing each of the 3*d* transition metals. That each of the central metals in these ions sits in an octahedral coordination environment provides an excellent illustration of the utility of the Kepert (1972 ▶) model and the inapplicability of the VSEPR model to *d*-block metal complexes. Closer examination of the [*M*(H_2_O)_6_]^*n*+^ structures across the 3*d* transition metals reveals that some possess nearly idealized O_*h*_ point-group symmetry, whereas others are distinctly *D*_4*h*_. Those with *d* ^9^ and high-spin *d* ^4^ configurations display the expected Jahn–Teller distortions. The Jahn–Teller effect can be readily illustrated to students by using the full CSD System to locate, *e.g.*, all CuO_6_ systems, retrieving the six Cu—O bond lengths for each substructure (using *ConQuest*) and then plotting those bond lengths as a histogram (*Vista*), as shown in Fig. 9 ▶. It is then easy to observe the very different distance ranges exhibited by the shorter equatorial Cu—O bonds (four per substructure) and the elongated axial bonds (two per substructure).

#### 4.2.2. Metal–carbonyl backbonding

**Figure 10:**
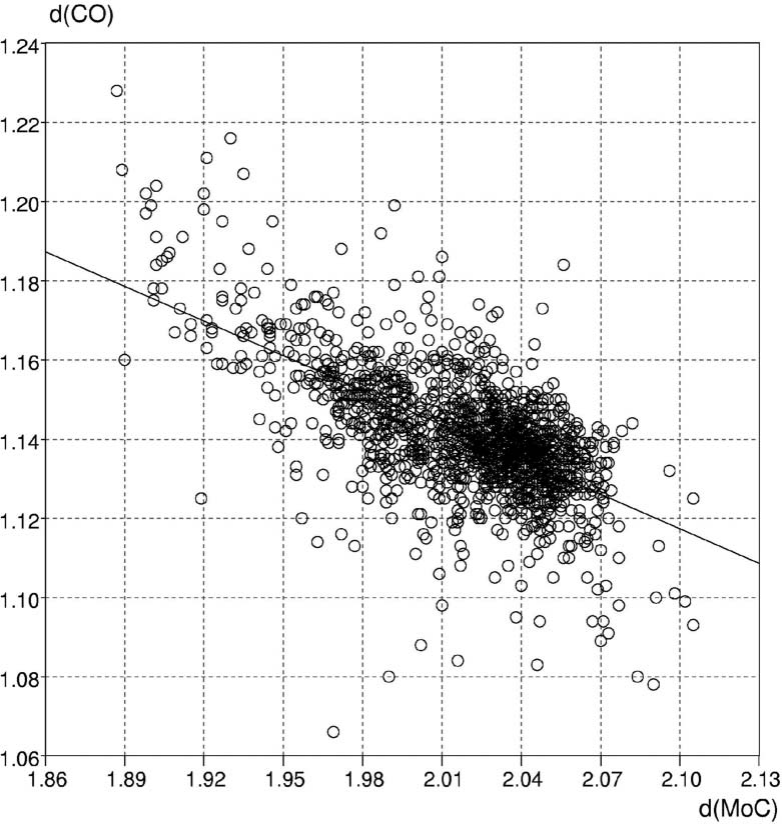
Metal–carbonyl π backbonding: plot of *d*(MoC) *versus* *d*(CO) for monodentate molybdenum carbonyl ligands.

The full database is an excellent resource for the exploration of π backbonding. For example, in Unit 7 of Table 3 ▶ the correlation between the Mo—C(carbonyl) distance, *d*(MoC), and the carbonyl bond distance, *d*(CO), is explored. The CSD contains over 35 000 entries with carbon monoxide coordinated to one or more transition metal atoms. *ConQuest* is used to carry out a three-dimensional search for CO bound to molybdenum to gather *d*(MoC) and *d*(CO) distances. The parameters from over 1400 monodentate molybdenum-bound carbonyl ligands are shown in the *Vista* scatterplot of Fig. 10 ▶, which clearly shows an overall linear relationship between carbonyl lengthening concomitant with Mo—C shortening, a feature consistent with modern π-backbonding models.

Concepts regarding π backbonding may be further explored in a wide range of types of organometallic complexes. A particularly nice example involves contrasting the structures of several transition metal complexes containing η^2^-bound alkyne ligands with the structure of a lanthanide complex containing a η^2^-bound 2-butyne ligand. In (η^2^-PhC CPh)_2_Pt (DPACPT10), (η^2^-PhC CPh)Pt(PMe_3_)_2_ (GACJAV), (η^2^-ClC CCl)Pt(PPh_3_)_2_ (PIYMUF) and (η^2^-F_3_CC CCF_3_)Pt(PPh_3_)_2_ (TPFYPT) coordination of the linear alkyne results in substantial bending of the *R*—C C bond angles, consistent with a bonding model whereby σ donation of ligand π electron density to the metal is accompanied by back donation of metal electron density into antibonding ligand orbitals resulting in formation of a metallocyclopropene. In stark contrast, Cp*_2_Yb(η^2^-MeC CMe) (FEKXOI) retains nearly linear Me—C C bond angles consistent with the electrostatic (as opposed to covalent) bonding observed for lanthanides.

#### 4.2.3. Reaction pathways and interconversions of metal geometry

**Figure 11:**
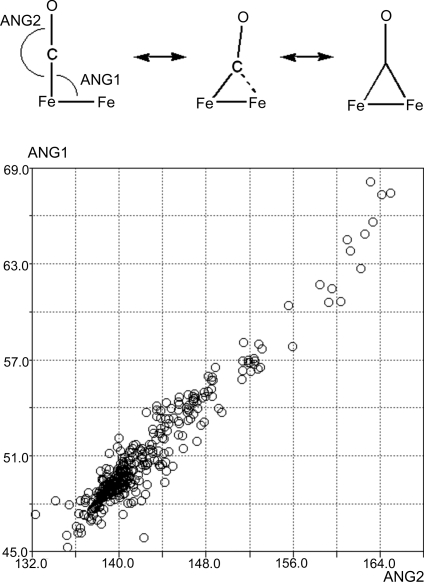
Scatterplot quantifying the change in geometry that occurs during carbonyl exchange in iron carbonyls.

The use of crystal structure information for the study of organic reaction pathways is described in §[Sec sec4.1.4]4.1.4. This principle, when applied to variations of geometry within metal coordination spheres, can be used to investigate the inorganic reaction mechanisms of ligand substitution and exchange. For example, the CSD contains numerous crystal structure determinations of iron carbonyl derivatives. These structures contain either a terminal carbonyl group, a carbonyl ligand that symmetrically bridges two Fe atoms or a semi-bridging carbonyl group where the ligand is bound asymmetrically. These bridged compounds may be considered as snapshots of the carbonyl exchange process. The scatterplot (Fig. 11 ▶) quantifies the change in geometry that occurs during carbonyl exchange. The carbonyl ligand proceeds through a bridging conformation in which the Fe—Fe—C angle (ANG1) becomes more acute and the Fe—C—O angle (ANG2) less linear. The series of crystal structures shown constitute a smooth continuum in which the Fe—Fe—C angle falls from *ca* 70 to 45° as the carbonyl group moves from the semi-bridging form through to the symmetrically bridged state. During this transition the Fe—C—O angle becomes less linear and the Fe—C distance shortens. Ideal relationships between parameters may be obtained by linear regression. Using these data, the trajectory of a carbonyl ligand during exchange between two iron centres may be plotted. The CO exchange process was originally studied using the structure correlation method by Crabtree & Lavin (1986 ▶).

**Figure 12:**
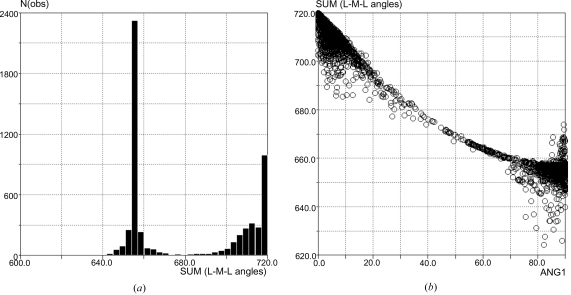
(*a*) The geometries adopted by four-coordinate transition metal complexes described using the sum of the four *cis*-*ML*_2_ angles and the two *trans*-*ML*_2_ angles. (*b*) Pathway for the interconversion between square-planar and tetrahedral geometries mapped using the sum of the *ML*_2_ angles *versus* the angle between the two *ML*_2_ planes.

Similarly, this approach can be used to explore the interconversion of alternative coordination geometries. The geometries adopted by four-coordinate transition metal complexes can be described using the sum of the four *cis*-*ML*_2_ and the two *trans*-*ML*_2_ angles. The resultant bimodal *Vista* distribution (Fig. 12 ▶ *a*) shows that four-coordinate metals have a tendency to adopt one of two geometries: square planar or tetrahedral. Structures with tetrahedral geometries can be found around 660° in the histogram (6 × 109.5°), while the square-planar structures are found close to 720° (4 × 90° plus 2 × 180°). However, the preferred square-planar and tetrahedral geometries can be affected by, for example, the nature of the substituents, and thus not all metal complexes have idealized conformations. A plot of the sum of the *ML*_2_ angles *versus* the angle between the two *ML*_2_ planes readily demonstrates this (Fig. 12 ▶ *b*). Although the plot shows that most four-coordinate complexes are either square planar (in the top left of the scattergram) or tetrahedral (bottom right), there are many database entries linking the two geometries resulting in a continuum. The structures that are neither square planar nor tetrahedral can be regarded as snapshots of transition states along the interconversion pathway.

Examples also exist which illustrate the sometimes subtle energetic difference between these two geometries. The crystal structure of dibromo-bis(benzyldiphenylphosphine)nickel(II) (DBBZPN) is an unusual example of an interallogon crystal structure. Two crystallographically independent Br_2_(Bn_3_P)_2_Ni molecules are present, and one contains the nickel in a square-planar coordination environment, while the other contains the nickel in a distinctly tetrahedral coordination environment.

#### 4.2.4. The isolobal analogy

In his Nobel Prize lecture *Building Bridges Between Inorganic and Organic Chemistry*, Hoffmann (1982 ▶) discussed the isolobal analogy, which compares the frontier molecular orbitals of traditional organic fragments with those of organometallic fragments, and elegantly illustrates how many, including some apparently complicated, organometallic molecules may be reduced to more simplistic but analogous complexes, and often to relatively simple organic molecules. This paper is an excellent case study for use in an advanced undergraduate inorganic chemistry course, since the isolobal analogy is a modern and useful model, and the manuscript is readily comprehensible by the typical target student audience. The principles and utility of the isolobal analogy are widely illustrated and defended in the paper through presentation of examples that can be found in the CSD. For example, the paper explores the similarities between the purely organic tetrahedranes, such as (Me_3_CC)_4_ (CUCZUP), and the tetrameric iridium carbonyl Ir_4_(CO)_12_ (FOJVEF). Visualizing these structures using *Mercury* will help students to obtain a clearer understanding of the examples, as well as to reinforce that this theoretical analogy is strongly supported by structural information obtained from known compounds.

#### 4.2.5. Crystal packing

Examples taken from the CSD may also be used to better illustrate concepts that are traditionally taught using more simplistic examples. This helps to mitigate the inadvertent teaching of misconceptions that will later need to be ‘unlearned’. Commonly, freshman chemistry students are taught the basics of solid-state packing through exclusive use of simple inorganic salts such as sodium chloride, caesium chloride, fluorite and zinc sulfide. Most of the traditional choices involve cubic lattices with anions occupying the primary special positions. This tends to suggest that most solid-state structures involve cubic lattices when, in fact, less than 0.5% of known structures pack in cubic arrays. Even more importantly, it promotes the misconception that atoms always lie on unit-cell corners, face centres, body centres and unit-cell edges. Consider the structure of the caesium salt of the C_60_^6−^ buckyball hexanion (FULLER). The structure clearly contains body-centred cubic packing; however, no atoms lie at the corners of the unit cell or at the centre of the unit cell. Rather, it is the centres of the C_60_^6−^ ions that are positioned at the corners and centre of the unit cell. One might also choose to illustrate that, while a face-centred cubic lattice might include atoms at the corners and face centres of unit cells (JUKPAG), corners and face centres may represent points in space about which molecules are arranged (GALGUV01), or all atoms and molecules may be well offset from these special positions. The same scenario may be described for a primitive cubic example like the structure of [Tl][Co(CO)_4_] (FUBZOR), and it is relatively easy, when visualizing with *Mercury*, to explore beyond just cubic examples.

### 4.3. Lessons from the literature

A number of the examples discussed in this paper have their origins in published analyses of crystal structure data, the most obvious example being the reaction pathway studies of Bürgi, Dunitz and co-workers discussed above (Bürgi & Dunitz, 1986 ▶, 1994 ▶). For many years, the CCDC has maintained a bibliography of major research studies that use the CSD and other CCDC products. This database is freely available and searchable *via* the CCDC website (CCDC, 2010*b* ▶) and contains a variety of CSD research applications which are likely to transfer rather well into the teaching environment. Clear examples are early papers by Dunitz and co-workers which studied the structural characteristics of carboxylic amides (Chakrabarti & Dunitz, 1982 ▶) and carboxylic esters (Schweizer & Dunitz, 1982 ▶), as well as work on the directional preferences of nonbonded atomic contacts by electrophiles and nucleophiles with divalent sulfur (Rosenfield *et al.*, 1977 ▶). Other key intermolecular studies include the analysis of directional preferences in hydrogen bonding to O-atom acceptors (Murray-Rust & Glusker, 1984 ▶) and proof positive of the existence of hydrogen bonds involving C—H donors (Taylor & Kennard, 1982 ▶). Some recent reviews in a database special issue of *Acta Crystallographica Sections B* and *D* are also useful in selecting potential teaching material. These reviews covered CSD applications in molecular inorganic chemistry (Orpen, 2002 ▶), the life sciences (Taylor, 2002 ▶), and organic and crystal chemistry (Allen & Motherwell, 2002 ▶).

## 5. Molecular symmetry and crystallographic symmetry

An understanding of the symmetry properties of molecules and crystals, and the inter-relationships between molecular and crystallographic symmetry, is fundamental to crystallography and is also central to many aspects of physics, chemistry, materials science and materials engineering. Individual symmetry elements are typically represented in text books by simple drawings (see, *e.g.*, Burns & Glazer, 1990 ▶; McKie & McKie, 1986 ▶), and for crystallographic point groups and space groups various graphical representations are commonly used (Hahn, 2005 ▶). Students can find these representations difficult to interpret in the abstract, whereas direct visualization of real structures by means of computer graphics can greatly aid the teaching of point-group and space-group symmetry at the undergraduate and graduate levels.

**Figure 13:**
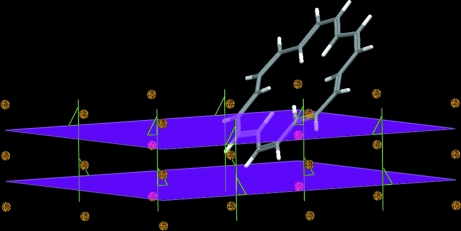
*Mercury* display of symmetry elements in (18)annulene.

The *Mercury* program (Macrae *et al.*, 2006 ▶) will display the space-group symmetry elements of a structure. Different graphics elements are used to denote, for example, inversion centres, rotation axes, screw axes, mirrors and glide planes. Thus, (18)annulene (teaching subset: ANULEN) displays *D*(6*h*) molecular symmetry, a point group that is relatively rare in the CSD. However, like most molecules belonging to a point group that encompasses inversion symmetry, it crystallizes on an inversion centre, in this case in the most popular space group *P*21/*c* for which the *Mercury* plot showing the relevant symmetry elements is shown in Fig. 13 ▶. Alternatively, *Jmol* (2010 ▶) now also provides a number of crystallographic symmetry display capabilities (Hanson, 2009*a* ▶,*b* ▶) applicable to CSD entries and other structures.

The relationship between molecular and crystallographic symmetry in CSD entries is the subject of a separate relational database called CSDSymmetry (Yao *et al.*, 2002 ▶). This database, built using Microsoft *Access*, is regularly updated and is freely available *via* the CCDC website (CCDC, 2010*d* ▶). The database contains information such as the molecular point group, crystallographic space group, *Z*, *Z*′ and the symmetry of the occupied Wyckoff positions for >400 000 unique CSD molecules. Auxiliary tables provide further information, such as the symmetry operators of the 230 space groups and the symmetry elements of the 38 point groups. CSDSymmetry can be interrogated with a wide variety of queries, for example ‘return all molecules with a mirror plane that are located on a crystallographic twofold axis’, thus allowing teachers to readily identify interesting molecules with which to exemplify symmetry concepts. CSDSymmetry has been surveyed by Pidcock *et al.* (2003 ▶) to obtain distributions of molecules over the different Wyckoff positions and to characterize some relationships between molecular and crystallographic symmetry.

**Figure 14:**
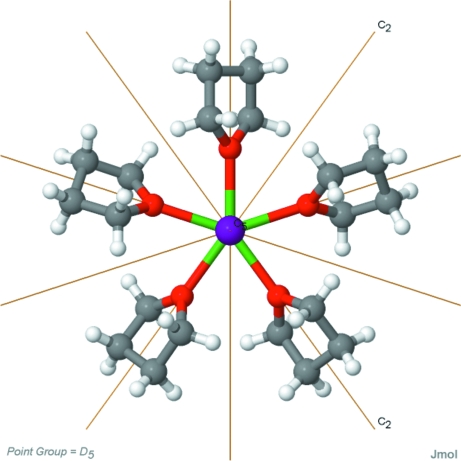
Display of symmetry elements in YbI_2_(THF)_5_ (THF is tetrahydrofuran) from Johnston’s (2009*b* ▶ ▶) symmetry gallery.

The definition of symmetry operations involves the concept of motion of an object: an object has a symmetry property when it can be brought into self-coincidence by an isometric motion (*i.e.* by a translation, rotation, mirror or inversion operation), and students can struggle to perform these mental operations on three-dimensional objects without actually observing them using models or computer graphics. This issue has been addressed by Johnston (2009*a* ▶) who has created a website (Johnston, 2009*b* ▶) containing resources designed to help students learn concepts of molecular symmetry and to help faculty at Otterbein College (Westerville, Ohio, USA) and elsewhere teach these concepts. A point-group symmetry tutorial guides students through all of the symmetry elements and operations using interactive displays and animations. Johnston has used prior knowledge and CSDSymmetry to assemble a symmetry gallery of 70 unique molecules, which is provided with an interactive and animated display of symmetry elements as illustrated (statically) in Fig. 14 ▶. The molecules are organized by point group, so educators can readily select examples to demonstrate particular symmetry elements. Additionally, a simple interface for searching CSDSymmetry by point group is provided. The site also contains a symmetry challenge section, incorporating a flow chart that details the process of determining the point group of a particular molecule, thus providing an interactive route for students to practice point-group determination.

## 6. Conclusion

The CSD is, of course, a crystallographic database, and has tremendous value to teachers of the subject in choosing examples of specific types of structure for more detailed study as part of a formal course. Issues connected with crystallographic and molecular symmetry are discussed in the main text, and it is worth noting that examples of all 230 space groups are represented in the database. Additionally, it is a simple matter to locate examples of disorder (in all its aspects), twinning, absolute configuration determination, neutron studies, structures determined by X-ray and neutron powder diffraction *etc.* However, the principal strength of the CSD is that it represents a vast and growing compendium of three-dimensional chemical structures, and it is this aspect, arguably, that resonates most with a broad constituency of chemical educators. It is for this reason that we have concentrated almost entirely here on the value of three-dimensional chemical structures in the teaching environment. Our observation in recent years is that a growing number of teachers of undergraduate chemistry courses are finding value in the crystallographic databases in general, and the CSD and PDB in particular. Not only does this activity introduce students to the crucial importance of crystallographic methods in furthering our understanding of three-dimensional chemistry in all its aspects, it also introduces them to the three-dimensional realities of the chemical world.

## Figures and Tables

**Table 1:** Teaching subset composition compared with statistics for the complete CSD *N*(subset) and *N*(CSD) are the numbers of structures in the teaching subset and complete CSD, respectively; %(subset) and %(CSD) are the relevant percentages.

Structure type	*N*(subset)	%(subset)	*N*(CSD)	%(CSD)
All structures	500	100.0	501857	100.0

Organic	331	66.2	215106	42.9
Transition metal–organic	161	32.2	266333	53.1
Main-group metal	86	17.2	31470	6.3

Organic structures				
Carbohydrates	8	1.6	5656	1.1
Nucleosides/nucleotides	6	1.2	1925	0.4
Amino acids and peptides	29	5.8	10383	2.1
Porphyrins/corrins	13	2.6	6158	1.2
Steroids	12	2.4	3839	0.8
Alkaloids	10	2.0	2678	0.5
Organic polymers	8	1.6	299	0.1

Metal–organic structures				
Any metal: three-coordinate	9	1.8	5527	1.1
Any metal: four-coordinate	37	7.4	58730	11.7
Any metal: five-coordinate	21	4.2	30537	6.1
Any metal: six-coordinate	52	10.4	83372	16.6
Any metal: seven-coordinate	10	2.0	13065	2.6
Any metal: eight-coordinate	10	2.0	6605	1.3
Any metal: nine-coordinate	6	1.2	3123	0.6
Any metal: ten-coordinate	2	0.4	1051	0.2
Any metal: (10+)-coordinate	5	1.0	463	0.1
π complexes	33	6.6	54235	10.8

Containing specific keywords				
‘Drug’ or ‘Activity’	42	8.4	15543	3.1
‘Polymorph’ or ‘Form’	80	16.0	16253	3.2

**Table 2:** Five teaching modules based entirely on use of the CSD teaching subset and *WebCSD*

Topic and objectives	Interactive activities
Unit 1: Aromaticity and the planarity of benzene	
To investigate the structural requirements for aromaticity. To understand the stability of benzene in terms of its molecular orbital description. To apply Huckels rule to predict whether or not certain compounds are aromatic.	Visualize a series of benzene and cyclooctatetraene derivatives, measure and compare the carbon–carbon bond lengths and planarity of the structures. Relate structural characteristics to the number of π electrons. Use findings to predict whether or not certain compounds are aromatic.

Unit 2: Ring strain and conformation	
To understand that angle strain can occur in cycloalkanes as a result of deviation from the ideal *sp*^3^ geometry and when neighbouring bonds are forced to be eclipsed (Pitzer strain). To be able to account for the conformations of three- to six-membered carbocycles in terms of the strain present. To explain why cyclohexane is essentially strain free.	Calculate angle strain for a series of fully saturated planar carbocycles. Measure the actual angle strain in cyclohexane by analysing structural data. Plot and compare calculated angle strain for planar rings with that measured in actual compounds. Visualize three- to six-membered carbocycles and account for the observed conformations.

Unit 3: Stereochemistry and chirality	
To recognize a stereogenic (chiral) centre in a molecule. To assign *R* and *S* configurations. To predict, identify and distinguish between enantiomers and diastereomers. To recognize a *meso* compound. To recognize other structural features that can give rise to chirality.	Compare two crystal structures of alanine and describe their relationship. Identify basic structural features that give rise to chirality. Describe the configuration of chiral centres in given molecules. Visualize and understand the relationship between the structures of threonine, ephedrine and tartaric acid. Examine further structures and recognize other features that can give rise to chirality, *e.g.* quadrivalent and tervalent chiral atoms, restricted rotation, and helicity.

Unit 4: VSEPR	
To investigate three-dimensional molecular shape. To understand factors that determine the preferred three-dimensional shape of specific molecules. To use the VSEPR model to predict three-dimensional molecular shape.	Examine the structures of di-, tri- and tetrachloromercury; determine the main factors that control the geometry adopted. Observe effects of lone pairs on geometry by examining [XeF_5_]^−^, water and dibromodimethylselenium. Apply the VSEPR model to predict the geometry of given molecules. Compare predictions with crystal structures and comment on how closely the observed bond angles agree with the expected ideal values.

Unit 5: Hapticity	
To investigate the concept of hapticity and learn its nomenclature. To examine the structural perturbations of ligands as a function of their hapticity.	Visualize given CSD structures and investigate the different modes of metal–carbon bonding. Relate nomenclature to structural features. Examine a series of structures and identify the hapticity of the organometallic ligands.

**Table 3:** Four teaching modules based on use of the complete CSD System

Topic and objectives	Interactive activities
Unit 6: Reaction intermediates – halonium ions	
To evaluate possible mechanisms for the electrophilic addition of Br_2_ to an alkene based on the stereochemistry of the products that are formed. To search the CSD for evidence of the existence of a cyclic bromonium ion intermediate. To account for the observed stability of the adamantylidene­adamantane­bromonium ion.	Investigate the stereochemistry of halogen addition by searching for evidence in the CSD that the cyclic bromonium ion actually does exist. Find other examples of halonium ions Explain the stability of the halonium ions found in the CSD.

Unit 7: Metal–carbonyl back bonding	
To search for molybdenum carbon monoxide complexes in the CSD using *ConQuest* and monitor the Mo—C and C=O bond lengths. To read the search results into *Vista* for further analysis. To rationalize the search results based on electron counting and orbital considerations.	Define a search for molybdenum carbon monoxide complexes. Define the relevant bond lengths of interest (the Mo—C and C=O bonds) and apply suitable constraints. Set the search running and analyse the results. Try and rationalize your observations.

Unit 8: Square-planar-to-tetrahedral interconversions at four-coordinate metals	
To determine the preferred geometries adopted by four-coordinate transition metal complexes by analysing the collected geometric data. To investigate some of the structures with non-idealized coordination geometries.	Search for four-coordinate transition metal complexes in the CSD and for each hit structure retrieve the values of the *L*—*M*—*L* angles (four ‘*cis*’ and two ‘*trans*’). Work out a single angular parameter to define the metal coordination geometry, and plot and analyse the data.

Unit 9: Molecular dimensions (basic)	
To determine the preferred value of an Sb—Cl bond length in SbCl_6_ by generating a bond-length distribution from CSD structures. To evaluate the precision of the results using statistical criteria. To examine the outliers in the bond-length distribution and attempt to distinguish those due to error from those of structural interest.	Search for SbCl_6_ ions in the CSD and retrieve all Sb—Cl bond lengths. What is the typical Sb—Cl bond length in hexachloro­antimony? How precise is this mean value? Investigate the outliers in the Sb—Cl bond-length distribution. Evaluate the structure that contains the longest observed Sb—Cl bond length.

**Table 4:** Mean C—C bond lengths (Å) in different chemical substructures determined using the CSD System *N*_obs_ is the number of observations retrieved from the CSD, σ_m_ (Å) is the s.u. of the mean value and σ_s_ (Å) is the s.u. of the sample.

Substructure	Mean *d*(C—C)	*N*_obs_	σ_m_	σ_s_
(C*sp*^3^)_2_—CH—CH—(C*sp*^3^)_2_	1.540	6301	<0.001	0.016
Cyclopropane	1.505	770	0.001	0.013
Cyclobutane (all bonds)	1.550	383	0.002	0.016
Cyclobutane (puckered)	1.547	318	0.002	0.015
Cyclobutane (planar)	1.564	65	0.002	0.011
